# Repetitive Biomimetic Self-healing of Ca^2+^-Induced Nanocomposite Protein Hydrogels

**DOI:** 10.1038/srep30804

**Published:** 2016-08-22

**Authors:** Jun Chen, Qiuchen Dong, Xiaoyu Ma, Tai-Hsi Fan, Yu Lei

**Affiliations:** 1 Department of Biomedical Engineering, University of Connecticut, Storrs, CT 06269, USA; 2 Department of Mechanical Engineering, University of Connecticut, Storrs, CT 06269, USA; 3 Department of Chemical and Biomolecular Engineering, University of Connecticut, Storrs, CT 06269, USA

## Abstract

Self-healing is a capacity observed in most biological systems in which the healing processes are autonomously triggered after the damage. Inspired by this natural behavior, researchers believed that a synthetic material possessing similar self-recovery capability could also be developed. Albeit various intrinsic self-healing systems have been developed over the past few decades, restriction on the biocompatibility due to the required synthetic conditions under extreme pH and with poisonous cross-linker significantly limits their application in biomedical field. In this study, a highly biocompatible nanocomposite protein hydrogel with excellent biomimetic self-healing property is presented. The self-healing protein gel is made by inducing calcium ions into the mixture of heat-induced BSA nano-aggregates and pristine BSA molecules at room temperature and under physiological pH due to the ion-mediated protein-protein association and the bridging effect of divalent Ca^2+^ ions. The as-prepared protein hydrogel shows excellent repetitive self-healing properties without using any external stimuli at ambient condition. Such outstanding self-recovery performance was quantitatively evaluated/validated by both dynamic and oscillatory rheological analysis. Moreover, with the presence of calcium ions, the self-healing behavior can be significantly facilitated/enhanced. Finally, the superior biocompatibility demonstrated by *in vitro* cytotoxicity analysis suggests that it is a promising self-healing material well-suited for biomedical applications.

Smart materials possessing rapid response to environmental changes have drawn increasing interests due to their unique biomimetic functions^1^,^2^. Self-healing is one of the most outstanding responsive properties observed in biological materials from single molecule (e.g., repair of DNA) to macroscopic level (e.g., closure and healing of injured blood vessels)^3^. It is a key feature for living organisms to survive and prolong the lifetime^4^. However, most of synthetic materials fail to heal themselves due to the rigid structure of their building blocks and the lack of chemical reformation mechanisms after the mechanical damage^5^. Inspired by nature, smart materials capable of mimicking a living system and healing the damage have attracted many creative studies. It has been demonstrated that self-healing materials are potentially capable for a wide spectrum of applications including drug delivery^1^,^6^, soft actuation^2^,^7^, biosensing^8^, tissue and organ repairing^9^,^10^, and shape memory functions^11^,^12^.

To realize self-healing property, different strategies have been explored over the past few decades. In one strategy, self-healing agents were first encapsulated in the self-healing system and were released upon the mechanical damage, and then formed covalent bond to heal the defect^3^,^13^,^14^,^15^. However, after running out of the limited amounts of healing agents, the self-healing process cannot be repeated in the same area^5^,^16^. Another self-repairing strategy relies on utilizing reversible chemical bonds or physical interactions. Such self-healing system can withstand repetitive damage due to their intrinsic stimuli-responsive self-healing properties. By employing the reversible but relatively strong dynamic covalent bonds, synthetic self-healing materials based on the equilibrium of bond breaking and reforming possess both structural and mechanical stability, which is well-suited for self-healing purpose^1^,^10^,^13^,^17^. Non-covalent bonds such as ionic interactions, hydrophobic interactions, π-π stacking and host-guest interactions were also employed to develop self-repairing systems^18^,^19^,^20^,^21^.

Besides synthetic materials, natural macromolecules have been reported in building self-healing systems to improve the biocompatibility. Numerous self-recovery systems reported in literature were made up by amphiphilic peptides, proteins, saccharides and/or their conjugated polymers^6^,^13^,^22^,^23^,^24^,^25^,^26^. However, for conjugated synthetic materials, poisonous cross-linkers and/or unfriendly healing conditions such as extreme pH or heating are generally required to accomplish self-healing, which significantly compromise their biocompatibility and bio-applicability. As a result, to realize the repetitive biomimetic self-healing system with good biocompatibility at benign biological conditions is still an unmet challenge yet critical for potential biomedical applications.

To address this challenge, for the first time here we report a novel self-healing system based on gelation of the mixture of thermally induced bovine serum albumin (BSA) protein nano-aggregates and fresh BSA molecules upon the addition of metal ions. BSA is a globular protein which could undergo heat-induced conformational changes leading to aggregation and gelation. The heat-induced gelation can be initiated by metal ions. With appearance of divalent cations such as Cu^2+^ and Zn^2+^, gelation of heat-induced BSA aggregates was achieved at room temperature^27^. To further improve the biocompatibility and to explore novel gelation strategy, benign calcium ions (Ca^2+^) were chosen as the substitute divalent ions to induce the gelation of heat-induced BSA nano-aggregates with the help of pristine BSA molecules. The as-developed self-healing system relying on albumin hydrogel was triggered by Ca^2+^ in the hydrogel at room temperature under physiological pH level, which has not been reported in the literature. The self-healing process is repeatable and a fully mechanical recovery was achieved without using any external stimuli at room temperature. Moreover, the presence of additional calcium ions (even at biological Ca^2+^ concentration level) could significantly promote and facilitate the self-healing behavior.

## Results

### Gelation of Ca^2+^ induced BSA hydrogel

**Figure 1:**
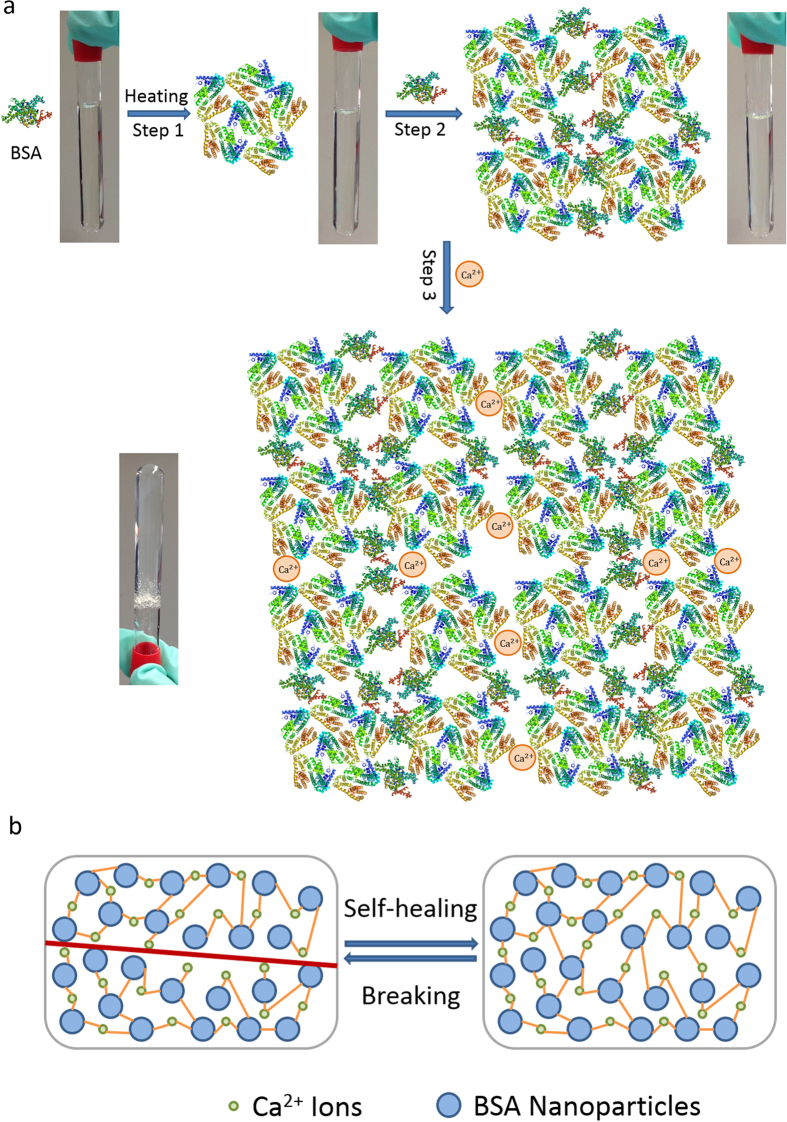
The self-healing protein hydrogel system. (**a**) The proposed gelation process. (**b**) The schematic of self-healing process in the as-prepared protein hydrogel.

**Figure 2:**
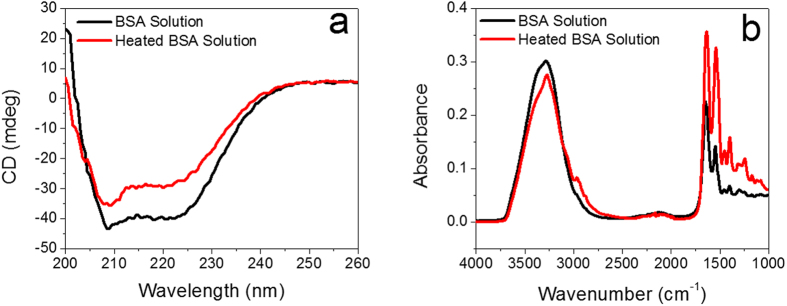
(**a**) CD spectrum of BSA solutions with and without heating. (**b**) FTIR spectrum of BSA solutions with and without heating.

As shown in Fig. 1a, the developed novel cold-gelation of BSA requires three steps: first, fibrillar BSA aggregates were obtained after thermally-induced unfolding of the native BSA protein and the as-prepared BSA nano-aggregates solution is transparent and homogenous due to the small size of BSA aggregates; then after cooling down to room temperature, further aggregation was achieved by adding fresh native BSA into the solution, in which the mixture remains transparent and homogeneous, and finally, gels were formed by inducing calcium ions into the mixture. During the heat-induced process, hydrophobic interactions, resulted from exposing hydrophobic groups originally buried in the core of native BSA protein, play a major role in the aggregation process in Step 1. Besides strong hydrophobic interactions, intermolecular hydrogen bonding and electrostatic interactions of the functional groups also contribute to intramolecular interactions after unfolding of the protein^28^. Such claim was supported by circular dichroism (CD) and FTIR spectra. CD spectra (Fig. 2a) show that the negative bands centered at 208 nm and 222 nm, which are characteristic of α-helix structure of BSA, decrease after heating at 80 °C for 30 min, indicating reduction of α-helix and formation of disordered conformations. The increase of peak intensity of both Amide I and Amide II in FTIR spectra (Fig. 2b) suggest an increased number of hydrogen bonds. By adding fresh and highly concentrated native BSA solution, excessive amount of functional groups in BSA fibrillary aggregates interact with native BSA protein molecules to form bigger aggregates. Finally, Ca^2+^ ions were added into the aggregate solution to shield the electrostatic charge present on the protein surface, bridging the spherical aggregates and leading to the gelation which was originally prevented in Step 2 due to relatively high electrostatic energy barrier. The divalent ions act as bridges between the negative charged groups on neighboring protein molecule to form a cross-linked network. The ion-mediated protein-protein association and the bridging effect of divalent Ca^2+^ ions provide an excellent strategy to develop biocompatible self-healing nanocomposite protein hydrogels without using any external stimuli at room temperature and physiological pH (Fig. 1b).

### Dynamic light scattering experiment

**Figure 3:**
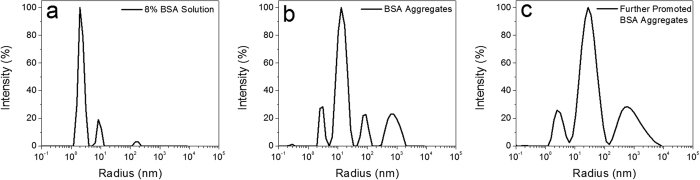
BSA size distributions. (**a**) BSA solution. (**b**) BSA solution heated at 80 °C for 30 min. (**c**) 10% BSA mixture of heated and fresh-prepared BSA solution.

To verify the hypothesis above and better understand the gelation mechanism, the size distributions of heat-induced protein aggregates were studied by dynamic light scattering (DLS). The size distributions in BSA solution before and after heating as well as the mixture of heated BSA solution and fresh native BSA solution at room temperature were compared. As shown in Fig. 3a, three groups of particles (difference in size) were observed in BSA solution before thermal treatment with a particle size of about 2, 8 and 200 nm, respectively. Majority of BSA in the solution before thermal treatment are single molecule with a size of few nanometers. A small portion of particles observed with larger size in solution is probably ascribed to the metastable clusters formed^29^. After incubation at 80 °C, the large particles with size of about 200 nm disappeared. Instead, four major classes of particles with size of 3, 13, 90, and 800 nm appeared, attributed to the thermal-induced aggregations (Fig. 3b). After mixing with highly concentrated fresh native BSA protein solution, a major species of particles with a size centered at about 30 nm and two other species at 2.5 and 600 nm (Fig. 3c) appeared, indicating that native BSA molecules can also interact with the heat-induced BSA aggregates and result in further aggregation or redistribution of the aggregates. The DLS study clearly demonstrates that highly concentrated native BSA molecules promote the aggregation of thermal-induced protein nano-aggregates. Therefore, the mechanical strength of the gel can potentially be enhanced by both increasing the protein concentration and the size of the building blocks.

To explore the dominance of interactions within the hydrogel matrix, 10% BSA hydrogels with 20 mM Ca^2+^ were incubated in various chemical environments. Supplementary Figure S1 shows that the calcium BSA hydrogels were stable in acidic (1 M HCl), physiological (20 mM PBS at pH 7.4), and EDTA-H_2_O (1.25% EDTA) conditions. Calcium BSA hydrogels immersed in basic condition (1 M NaOH) were dissolved within 1 h. Also, samples in 8 M urea were completely dissolved within 24 h, indicating that non-covalent and probably hydrogen bonding is the primary mechanism for the cross-linkages. Additionally, hydrogel samples submerged in 10% SDS turned into transparent and shrunk by 50% in volume, suggesting that hydrophobic interactions also play a role in maintaining the gel network.

### Dynamic rheological analysis

**Figure 4:**
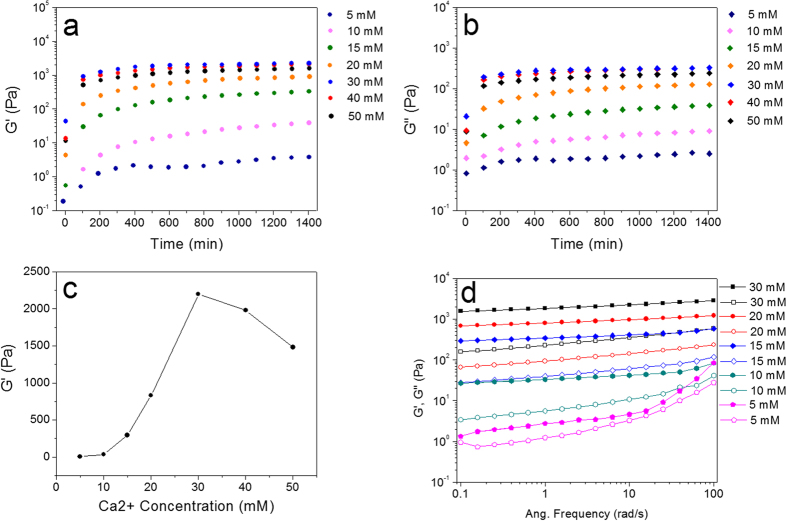
Changes of storage modulus G′ (**a**) and loss modulus G″ (**b**) at 1 Hz versus time for protein hydrogel forming under a constant total BSA concentration (10 wt%) and different Ca^2+^ concentrations at 25 °C. (**c**) G′ versus Ca^2+^ concentration at time = 1200 min. (**d**) G′ and G″ versus oscillation frequency at various BSA concentrations.

To characterize the effect of Ca^2+^ concentration on the viscoelastic properties of the Ca^2+^-induced protein hydrogel, dynamic rheological measurements were systematically conducted. The storage modulus (G′) and loss modulus (G″) of different BSA hydrogels induced by different Ca^2+^ concentrations were presented (Fig. 4). Seven pairs of G′ (Fig. 4a) and G″ (Fig. 4b) at a frequency of 1 Hz for a constant protein concentration (10 wt%) were plotted as a function of time after addition of various Ca^2+^ concentrations. All samples have exhibited solid-like property only a few minutes after Ca^2+^ addition. Both G′ and G″ significantly increased with the increase of Ca^2+^ ions up to 30 mM, and then slightly decreased when Ca^2+^ increased above 30 mM. The G′ is about an order of magnitude larger than G″ when the Ca^2+^ concentration reached 10 mM (Fig. 4d), indicating that a strong hydrogel was formed via non-covalent bonding or bridging through Ca^2+^. In Fig. 4c, the elastic moduli obtained at time = 1200 min after the addition of Ca^2+^ were presented as a function of Ca^2+^ concentration. The elastic modulus increases with the increase of Ca^2+^ concentration up to 30 mM, suggesting a quick and increased association between Ca^2+^ and negatively charged groups on the BSA aggregates and native BSA molecules. Furthermore, the SEM images of lyophilized BSA hydrogels (Supplementary Figure 2a to 2d) with different Ca^2+^ concentrations (up to 30 mM) show that the hydrogel network appears to be more compact and homogenized as Ca^2+^ concentration increases, implying that the enhanced mechanical strength of the hydrogel (up to 30 mM Ca^2+^) is due to the rearrangement of the microstructure and the increase of cross-links. However, as Ca^2+^ concentration increases over 30 mM, the elastic modulus starts to decrease gradually. This could be attributed to the fact that the charge present on the surface of the protein aggregates and native fresh BSA molecules might be fully shielded by excessive amount of Ca^2+^ ions, leading to a formation of larger aggregates as a relatively coarse and weaker structure with lower elastic modulus.

### Self-healing performance of calcium BSA hydrogels

**Figure 5:**
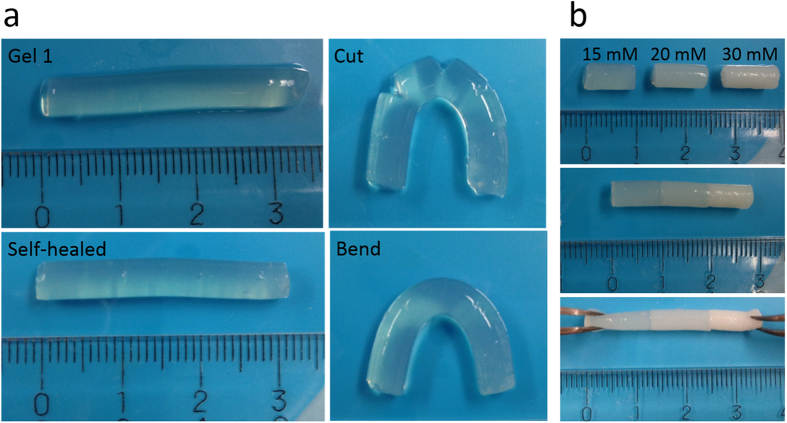
Photographs that demonstrate the self-healing behavior of the protein hydrogel at room temperature. (**a**) 10% BSA hydrogel with 12.5 mM Ca^2+^ at original, cut, self-healed, and bend states. (**b**) Self-healing of 10% BSA hydrogels with different Ca^2+^ concentrations (15, 20, and 30 mM).

The self-healing capability of the protein hydrogel with different Ca^2+^ concentration was investigated at room temperature. All BSA solutions were dissolved in a 20 mM potassium phosphate buffer (PBS) at pH 7.4. Highly concentrated native BSA solution was mixed with cooled low concentrated thermal-treated BSA solution to a final concentration of 100 mg/mL (10 wt%). Then, calcium chloride (CaCl_2_) solution was introduced to the 10 wt% protein aggregates solution for cross-linking and the mixture was maintained in a closed tubing for 24 h to allow the reaction to reach equilibrium, and then gels with different Ca^2+^ concentrations were obtained with predefined size and shape of the tubing. First the biomimetic self-healing property without using any external stimuli at ambient conditions was demonstrated by the healing through several cuts on the hydrogel rods. As shown in Fig. 5a, gel rods were prepared and cut with a razor at several places, which could be easily observed after bending the gel. The cutting surfaces were then brought together and kept in contact in a closed tube to avoid water evaporation from the hydrogel at room temperature. After 24 hours, self-healing of the cuts was observed as there is no rupture any more when bending the hydrogel and the scars were barely seen. The self-healed gel was cut again at the scars and then was put together closely to repeat the self-healing process. The result demonstrates the self-healing property of the calcium-induced BSA hydrogel that can fully achieve a repetitive biomimetic self-healing process. This self-healing property among different hydrogel segments prepared with different Ca^2+^ concentrations was also demonstrated in Fig. 5b. The gel rods with three Ca^2+^ concentrations (15, 20, and 30 mM) were prepared and cut into same length which were then brought together and kept with the surfaces fully in contact. After 24 hours, the three pieces have been coalesced with clear boundaries but no rupture occurred when manually stretching the samples, demonstrating that multiple hydrogel segments with various Ca^2+^ concentrations all possess self-healing capability.

**Figure 6:**
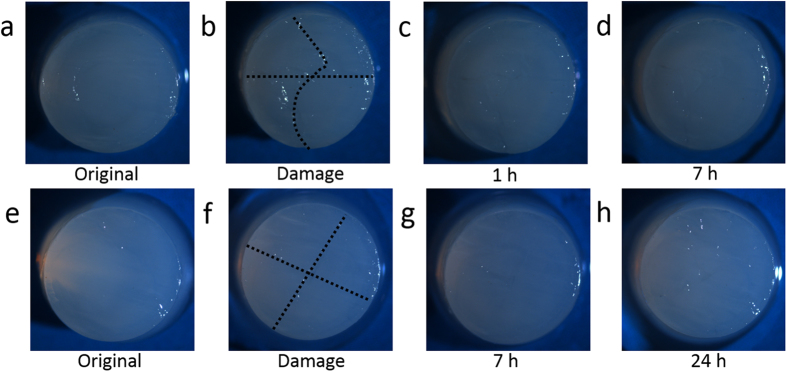
Photographs of the self-healing process of the protein hydrogel at room temperature. (**a**–**d**) are calcium-induced BSA hydrogel self-healed in PBS buffer with physiological levels of Ca^2+^. Images are taken at different time intervals: (**a**) original, (**b**) immediate after damage, (**c**) after 1 hr, and (**d**) after 7 hrs of self-healing; (**e**–**h**) are calcium-induced BSA hydrogel self-healed in PBS buffer without Ca^2+^. Images are taken at different time intervals: (**e**) original, (**f**) immediate after damage, (**g**) after 7 hrs, and (**h**) after 24 hrs of self-healing.

The efficiency of self-healing of the gel in scar recovery with and without calcium ions was also studied. It can be seen that calcium-induced BSA hydrogel in PBS buffer with physiological Ca^2+^ concentration (2.6 mM) was self-healed much faster than the hydrogel healed in PBS buffer without Ca^2+^ (Fig. 6). After 7 hrs of self-healing at room temperature, the sample in the presence of physiological Ca^2+^ was completely repaired and the scars from damages were barely seen, while the sample without Ca^2+^ showed relatively low self-healing rate and the scars were still obvious after 7 hrs of self-healing. After 24 hrs, the sample in PBS buffer without Ca^2+^ was completely repaired too. These results show that although not necessary for self-healing, the presence of Ca^2+^ can significantly promote self-healing of the hydrogels even at a very low physiological Ca^2+^ concentration (2.6 mM).

**Figure 7:**
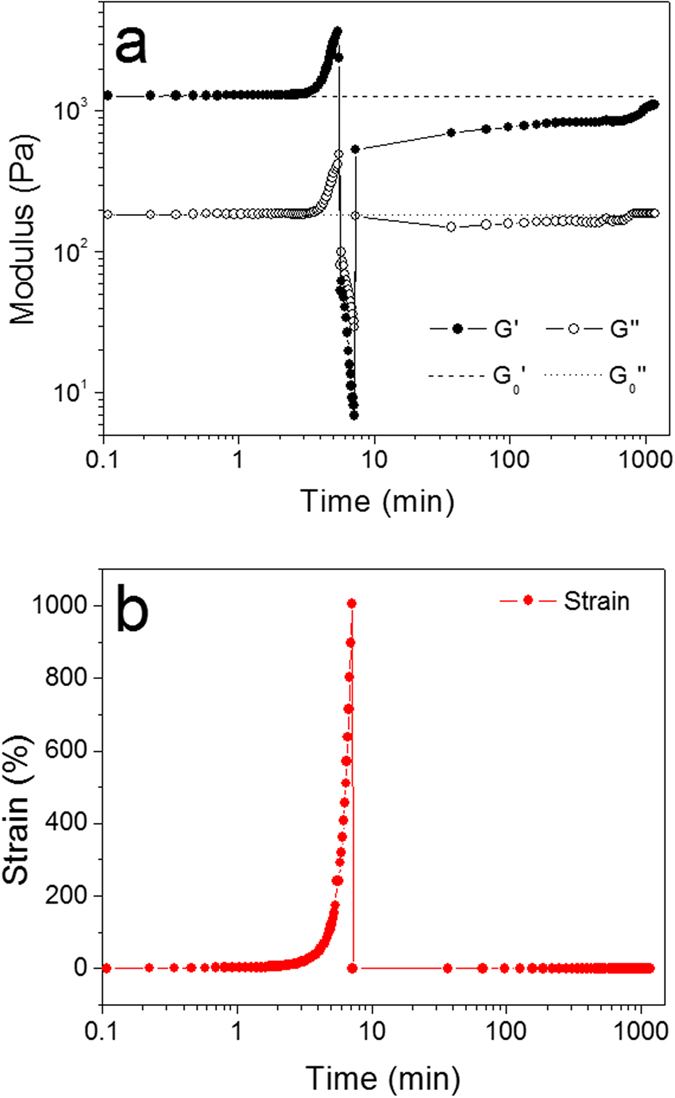
Transient properties of self-healing of hydrogel after fracture. (**a**) The elastic modulus 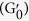 and loss modulus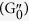 shown in dashed and dotted lines, respectively, are the reference moduli for the hydrogel before facture, the gel was fractured under a large strain and then G′ and G″ at 1 Hz and 1% strain were monitored. (**b**) The corresponding strain variation.

Furthermore, oscillatory rheological analysis was used to quantify the self-healing property of the hydrogel. The hydrogel was fractured first by increasing the shear strain to 1000%, then the storage modulus (G′) and loss modulus (G″) of the hydrogel were recorded with time at 1% low strain at oscillatory frequency 1 Hz. Figure 7a shows the plots of the moduli (G′ and G″) versus time and the corresponding strain variation is presented in Fig. 7b. At the fracture point both G′ and G″ decrease dramatically, especially the G′, indicating the fracture of the protein hydrogel under a high shear load and suddenly into an almost unstressed state. After the high load was removed and strain re-adjusted to 1%, the viscous dissipation G″ quickly recovered to about 200 Pa and basically remained the same for the rest of the process, but G′ quickly recovered to about 500 Pa, gradually increased to approximately 1 kPa and approached the original G′ over 24 hrs. The calcium protein hydrogel has recovered 55% of its elastic microstructure, responsible for about 700 Pa of its original storage modulus after 30 min, revealing the rapid mechanical recovery of the fractured hydrogel structure without any external stimuli at room temperature. Additionally, the self-healing capability of the calcium BSA hydrogel was evaluated by tensile tests. Supplementary Figure S3 shows the breaking stress of BSA hydrogel in three conditions: original (hydrogel before breaking), self-healing without adding anything at room temperature for 24 hrs, and self-healing with the presence of physiological Ca^2+^ concentration for 7 hrs, respectively. In the case with added Ca^2+^, the self-healed hydrogels ruptured in the bulk region rather than at the contact surfaces, indicating that a strong healing took place at the coalesced interface. Both self-healed samples were able to endure 100% of the breaking stress of the original gels, demonstrating the fully recovered hydrogel and that the appearance of calcium ions can accelerate such recovery.

### Biocompatibility analysis by *in vitro* cytotoxicity test

**Figure 8:**
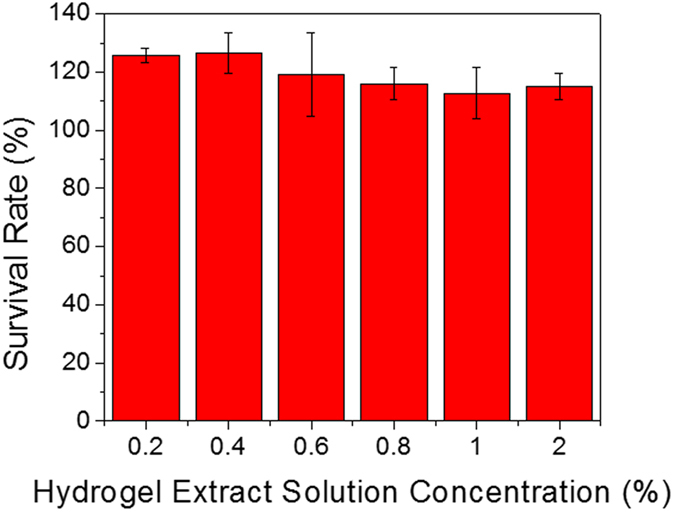
Cytotoxicity evaluation of the calcium-induced BSA hydrogel.

The biocompatibility of the sample was tested via the cell viability of carcinomic human alveolar basal epithelial cells A549 in presence of calcium-induced BSA hydrogel. Lyophilized samples were immersed into Dulbecco’s Modified Eagle’s Medium (DMEM) for 24 hours at 37 °C to yield a 2% (w/v) hydrogel extract solution. Then the hydrogel extract solution was diluted to different concentrations for cell culture. It shows that there is almost no interference with the presence of the hydrogel to cell viability even when the concentration of the hydrogel extract solution reached to 20 mg/mL (Fig. 8). More interestingly, the presence of hydrogel extract solution facilitates growth of cancer cells as all survival rates are over 110% compared with the control. Considering that albumin is the most abundant protein in blood plasma and calcium is required for Vitamin D absorption and bone regeneration in human body, the calcium-induced BSA hydrogel should possess high biocompatibility, corroborated by the cytotoxicity test. The data of cytotoxicity test suggest the potential use of this protein hydrogel in *in vivo* applications.

## Discussion

Self-healable protein hydrogels with superior biocompatibility were fabricated in this study. This protein hydrogel is able to be self-healed without any external stimulus at room temperature and under physiological pH, which are optimal for biomedical application. In addition, the presence of this material promoted cell growth rather than hampered, suggesting its superior biocompatibility and potency in applications such as tissue engineering, cell culture and drug delivery.

Heat-induced BSA nanoparticles and pristine BSA molecules were utilized as the building blocks in this novel system. After inducing calcium ions into the mixture of thermal-induced protein aggregates and pristine BSA molecules, BSA hydrogels were generated at room temperature without appearance of any traditional cross-linkers. The as-prepared novel nanocomposite protein hydrogels exhibited excellent self-healing behaviors. Specifically, fully recoveries were not only observed for the protein hydrogel prepared with the same Ca^2+^ concentration, but also demonstrated for multiple hydrogel segments prepared with different Ca^2+^ by simply keeping contact of the cutting surfaces at room temperature without any external stimuli. Besides, scar recoveries of the hydrogels were also tested, showing that appearance of calcium ions greatly promoted the healing processes though it is not necessary. The self-recovery performance was quantitatively evaluated by both dynamic and oscillatory rheological analysis. This repeatable and effective self-healing process without using any external stimuli at room temperature is accomplished primarily due to the divalent ions bridging effect, as the presence of calcium ions greatly promoted the self-healing process even at a very low concentration (e.g., physiological level of 2.6 mM). Without presence of Ca^2+^ ions, recovery of the hydrogels may rely on the dynamic breaking and re-bonding of divalent calcium ions to neighboring proteins aggregates.

Besides self-healing performance, superior biocompatibility of this system has also been demonstrated using *in vitro* cytotoxicity test. Even though numerous self-recovery systems have been developed in the past decades, intrinsic self-healing materials are barely utilized in any practical applications, especially in biomedical field. Dynamic covalent bond based materials usually require pH besides neutral condition to promote the self-healing performance^10^,^30^. A self-healing BSA hydrogel regulated by glucose oxidase and catalase was reported by Gao *et al*. possess 100% extent recovery capability^13^. However, biohazardous glutaraldehyde was used as the cross-linker and responsible for self-healing performance. A biocompatible self-healing hydrogel fabricated by cross-linking of alginate and calcium ions was demonstrated as a spatiotemporal controlling drug release system with applying ultrasound, which is elegant^6^. However, without the presence of Ca^2+^, alginate-Ca^2+^ hydrogel cannot be self-healed. That may limit its broad applications. Instead, albumin as the most abundant protein in circulatory system is able to serve as the low-cost, mass-produced substrate without further purification requirement. As a result, all these unique features of the self-healing calcium BSA hydrogel system suggest its potential application as highly biocompatible material in biological repairing as well as possibly for *in situ* drug delivery.

## Methods

### BSA hydrogels and analysis of self-healing

In this study, transparent and homogenous BSA nanoparticles solution was firstly fabricated by thermal treatment method. Then their further aggregation was obtained by adding fresh high concentrated BSA protein solution. Finally, gelation was triggered by inducing Ca^2+^ ions to the 10% protein nanoparticle mixture (w/v). BSA hydrogels with different Ca^2+^ concentrations were prepared by adjusting the amount of induced ions. Self-healing performances of protein hydrogels were observed after keeping the cut surfaces in contact for 24 h at room temperature. Quantitative evaluation was conducted by using AR-G2 rheometer. Briefly BSA nanoparticle and pristine BSA mixture solution with induced Ca^2+^ was firstly loaded onto the objective table to form hydrogel and then stabilized for 24 h. Then destruction of the hydrogel was obtained by increasing the shear strain to 1000% and self-healing process can be monitored immediately upon decreasing the stain to 1% after the destruction.

### Characterization of the BSA gelation

The formation of heat-induced protein aggregate nanoparticles was investigated by circular dichroism (CD) and Fourier transform infrared spectroscopy (FTIR). BSA powder was dissolved into potassium phosphate buffer at pH 7.4 and then incubated at 80 °C for 30 min before measurement. BSA solutions were utilized for FTIR measurement without dilution, and diluted by 2400-fold for CD measurement.

Dynamic light scattering (DLS) measurements were conducted using an ALV compact goniometer system (Germany) equipped with a 22 mW He-Ne laser source tuned at 632.8 nm. To demonstrate the presence of protein aggregates after thermal treatment and the further size changes after adding fresh BSA, a series of intensity scattering data were collected after BSA solutions were prepared, incubated at 80 °C for 30 min, and then mixed with highly concentrated fresh protein solution. The data on the size distribution of the aggregates in solution were obtained by ALV software through CONTIN procedure.

To explore the dominance of interactions within the hydrogel matrix, 10% BSA hydrogels prepared with 20 mM Ca^2+^ were incubated in various chemical environments. Hydrogel precursor solution was kept into a closed template at room temperature for 24 h after induction of calcium ions. The hydrogel sample was then cut to small cylindrical pieces with diameter of 5 mm and thickness of 3 mm, and immersed in different chemical environments including acidic (1 M HCl), basic (1 M NaOH), physiological (20 mM PBS at pH 7.4), 8 M urea, 10% SDS and EDTA-H_2_O (1.25% EDTA) conditions to study the morphology change with time.

### Morphology of the protein hydrogels

Morphological characteristics of the calcium protein hydrogels were explored by JEOL 6335 Field Emission Scanning Electron Microscopy (FESEM). All samples were frozen in liquid nitrogen and lyophilized for 24 h to maintain the porous structures, then sputter-coated with Au/Pd alloy before imaging at an acceleration voltage of 10 kV.

### Measurement of viscoelastic properties of the protein hydrogels

Mechanical properties of the protein hydrogels with different Ca^2+^ concentrations were explored by dynamic rheological measurements. AR-G2 rheometer (TA Instrument) was used for all rheological tests. 300 μL of protein nanoparticle and pristine BSA solution was well mixed with Ca^2+^ at different concentrations for the measurements. After stabilized for 24 h, frequency sweep was carried out for each calcium BSA hydrogel. The tests of storage modulus G′ and loss modulus G″ were carried out with a parallel plate (20 mm in diameter) at 25 °C and the oscillatory test was operated at 1 Hz.

### Cytotoxicity test of the protein hydrogel

*In vitro* assay was used to assess the cytotoxicity of the calcium BSA hydrogel on human lung carcinoma A549 cells. Protein hydrogels with 20 mM Ca^2+^ were lyophilized after freezing by liquid nitrogen for 24 h to evacuate all water in the matrix. And then lyophilized samples were submerged in Dulbecco’s Modified Eagle Medium (DMEM) and incubated at 37 °C for 24 h. A 2% (w/v) hydrogel extract solution was yielded and diluted to different concentrations (0.2, 0.4, 0.6, 0.8, and 1%) with DMEM. Cells were seeded in 96-well microplates with a number density of 5 × 10^4^ mL^−1^ in 100 μL suspension, then the cell medium was removed and cultured with 0, 0.2, 0.4, 0.6, 0.8, 1, and 2% hydrogel extract solution at 37 °C for 24 h. After the hydrogel extract replaced by fresh medium, Cell Counting Kit-8 (CCK-8) was added to each well and incubated for 1 h. Finally, absorptions at 450 nm was measured by a microplate reader and the cell survival rate was calculated by (A_sample_ − A_blank_)/(A_0_ − A_blank_) × 100%.

## Additional Information

**How to cite this article**: Chen, J. *et al.* Repetitive Biomimetic Self-healing of Ca^2+^-Induced Nanocomposite Protein Hydrogels. *Sci. Rep.* **6**, 30804; doi: 10.1038/srep30804 (2016).

## Supplementary Material

Supplementary Information
