# Supplementary material for: Repetitive Biomimetic Self-healing of Ca2+-Induced Nanocomposite Protein Hydrogels
Source: Sci Rep. 2016 Aug 22;6:30804. doi: 10.1038/srep30804 (PMC4992862; doi:10.1038/srep30804)
Supplement: Supplementary Information [file srep30804-s1.pdf]

Supplementary Information

Repetitive Biomimetic Self-healing of Ca^{2+}-Induced Nanocomposite Protein Hydrogels

Jun Chen[1], Qiuchen Dong[1], Xiaoyu Ma[1], Tai-Hsi Fan[2], and Yu Lei[1, 3*]

[1]Department of Biomedical Engineering, University of Connecticut, Storrs, CT 06269, USA

[2]Department of Mechanical Engineering, University of Connecticut, Storrs, CT 06269, USA

[3]Department of Chemical and Biomolecular Engineering, University of Connecticut, Storrs, CT 06269, USA

*Corresponding author

1. Calcium BSA hydrogel in different chemical environment

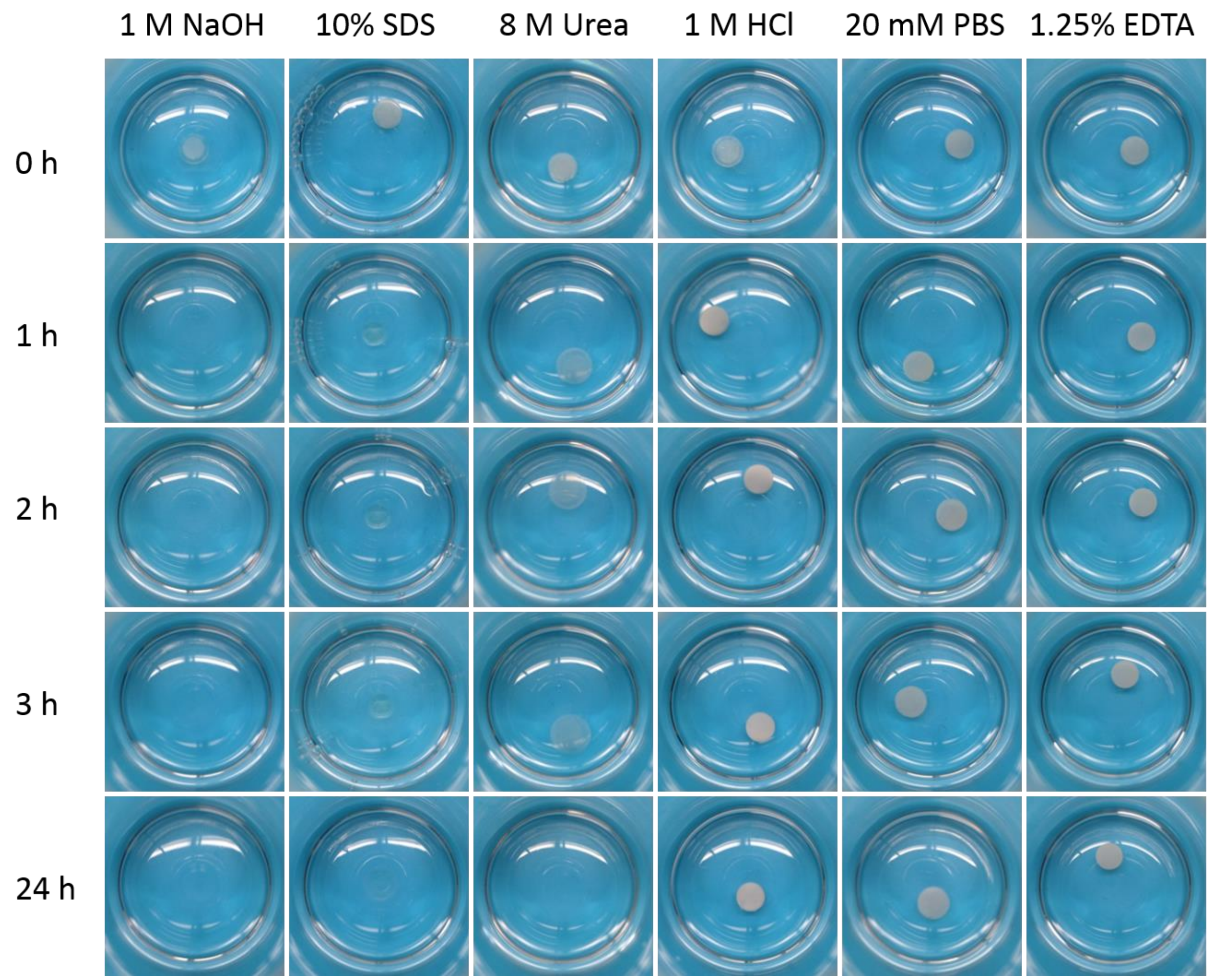

Supplementary Figure S1 | Time-dependent optical images of the as-prepared protein hydrogels in different chemical environment.

2. SEM images of calcium BSA Hydrogel

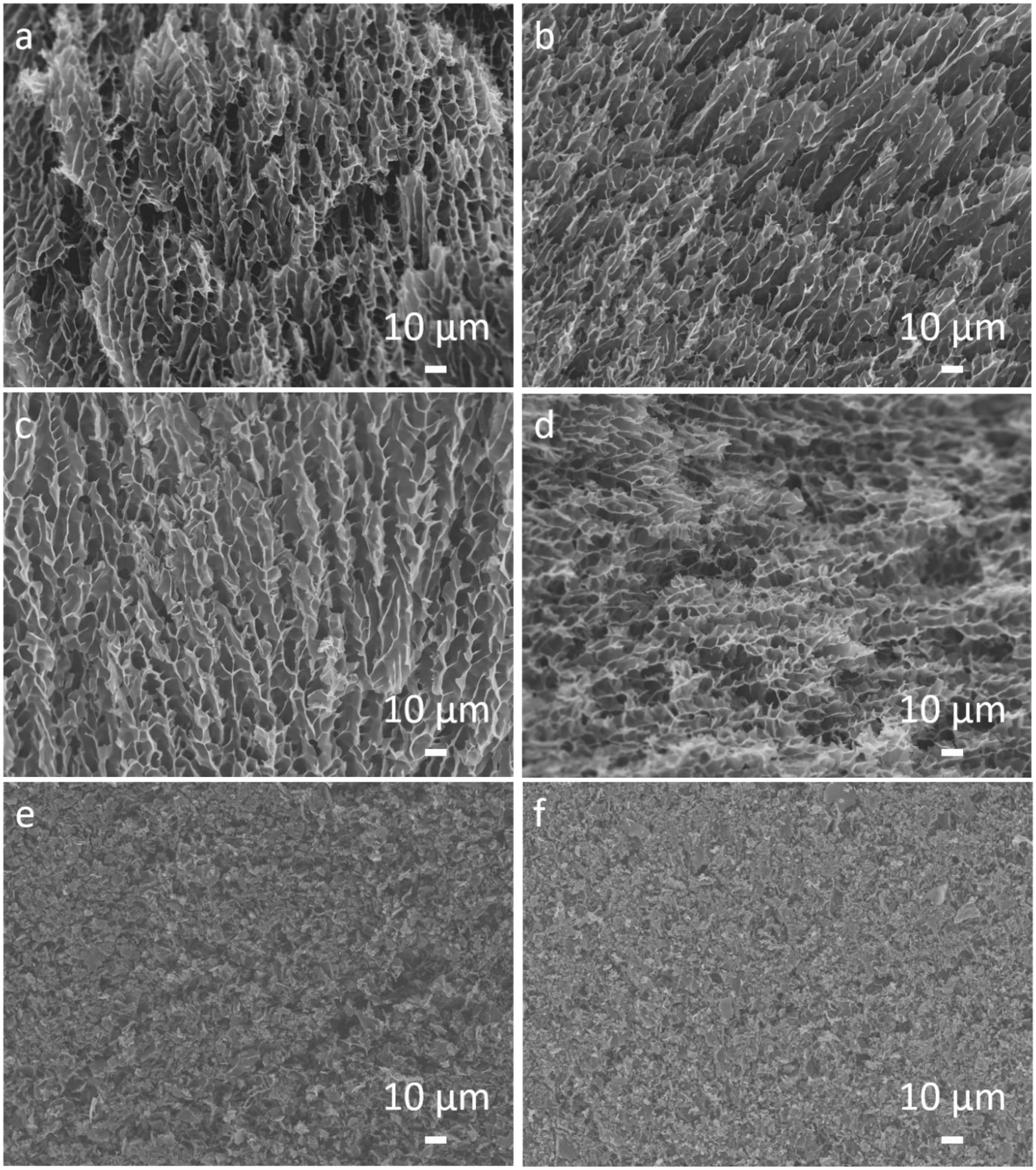

Supplementary Figure S2 | (a to f) SEM images of lyophilized calcium BSA hydrogels with 10, 15, 20, 30, 40, and 50 mM of Ca^{2+}, respectively.

3. Tensile test for quantitative self-healing evaluation

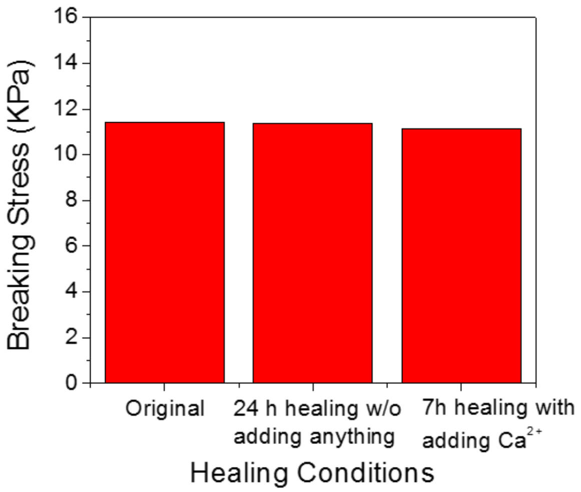

Supplementary Figure S3 | Breaking stress of 20 mM Ca^{2+}induced 10% BSA hydrogel: original, healed for 24 hrs at room temperature without adding anything, and healed with appearance of physiological Ca^{2+} concentration for 7 hrs at room temperature, respectively.
